# Urban hierarchy and spatial diffusion over the innovation life cycle

**DOI:** 10.1098/rsos.211038

**Published:** 2022-05-04

**Authors:** Eszter Bokányi, Martin Novák, Ákos Jakobi, Balázs Lengyel

**Affiliations:** ^1^ ELKH Centre for Economic and Regional Studies, Agglomeration and Social Networks Lendület Research Group, Budapest 1097, Hungary; ^2^ Corvinus University of Budapest, Laboratory for Networks, Technology and Innovation, Budapest 1093, Hungary; ^3^ ELKH Wigner Research Centre for Physics, Budapest 1121, Hungary; ^4^ Department for Regional Science, Eötvös Loránd University, Budapest 1117, Hungary; ^5^ ELKH Research Centre for Astronomy and Earth Sciences, Spatial Big Data Lab, Budapest 1121, Hungary

**Keywords:** diffusion, innovation, social networks, hierarchical diffusion, neighbourhood diffusion

## Abstract

Successful innovations achieve large geographical coverage by spreading across settlements and distances. For decades, spatial diffusion has been argued to take place along the urban hierarchy. Yet, the role of geographical distance was difficult to identify in hierarchical diffusion due to missing data on spreading events. In this paper, we exploit spatial patterns of individual invitations sent from registered users to new users over the entire life cycle of a social media platform. We demonstrate that hierarchical diffusion overlaps with diffusion to close distances and these factors co-evolve over the life cycle. Therefore, we disentangle them in a regression framework that estimates the yearly number of invitations sent between pairs of towns. We confirm that hierarchical diffusion prevails initially across large towns only but emerges in the full spectrum of settlements in the middle of the life cycle when adoption accelerates. Unlike in previous gravity estimations, we find that after an intensifying role of distance in the middle of the life cycle a surprisingly weak distance effect characterizes the last years of diffusion. Our results stress the dominance of urban hierarchy in spatial diffusion and inform future predictions of innovation adoption at local scales.

## 1. Introduction

Fuelled by the recognition that innovation drives economic progress [1,2], the adoption dynamics of new technologies and products over their life cycle have been analysed and modelled from the middle of the twentieth century [3,4]. Griliches [5] quickly made geography an important field of this discussion by demonstrating that adoption is faster in the proximity of the original location. Later, Hägerstand [6] modelled spatial diffusion with a special focus on cities; he distinguished categories of hierarchical (from a larger to a smaller city) and neighbourhood (determined by geographical proximity) processes. This classic work was applied in a wide range of contexts including the diffusion of ideas [7], fashion [8], culture [9] or e-shopping [10]. The mixture of hierarchical and neighbourhood diffusion mechanisms are thought to resemble routing behaviour in social media [11] such that spreading initially occurs over great distances between large cities but becomes more and more local when it reaches smaller towns. However, the separation of hierarchical versus neighbourhood diffusion is still problematic, because large cities are typically surrounded by smaller towns [12], which demands further analysis with data on spreading events.

More recently, adoption dynamics in social networks have received growing attention [13,14]. In this stream of literature, diffusion is modelled as a complex contagion process, in which the probability of individuals’ adoption increases as network neighbours adopt [15]. This model can predict the online spreading of behaviour and the diffusion of online products at the scale of countries well [16–18]. However, local predictions of the complex contagion framework still suffer from systemic biases [19]. City size and distance from the origin have remained major confounding factors in modelling spatial adoption [20] and still limit marketing applications at local scales [21].

In this paper, we aim to better understand the spatial diffusion of innovations by separating the role of urban hierarchy and geographical distance. This is done by analysing a unique database retrieved from a social media platform called iWiW, the most popular online social network in Hungary in the pre-Facebook era, adopted by 30% of the total population. We can trace the platform’s spatial diffusion through the dynamics of geolocated platform registrations, and the data also allows us to observe each spreading event—that is, accepted invitations registered users sent to new users—over the whole life cycle of the product from 2002 to 2012. Thus, we can reconstruct the direction of flows with unprecedented spatial granularity, scale and time horizon.

We demonstrate the problems of hierarchical versus neighbourhood diffusion categorization by showing their overlaps. In the middle of the life cycle, when adoption accelerates, the most probable spreading path across towns form long chains signalling that hierarchical diffusion emerges across the full spectrum of towns. In this period, large size differences between source and target towns of spreading events coincide with large shares of invitations sent very small distances in agglomeration areas where invitations tend to cascade from large cities to neighbouring small towns.

To disentangle hierarchical and neighbourhood diffusion, we estimate the number of invitations sent between pairs of towns by years with zero-inflated negative binomial regressions, in which the population sizes of the source and the target towns, their combination, and the distance between them are the explanatory variables. This framework allows us to separate the single effect of city size and distance and also consider their joint effect. By considering the direction of flows, we discover that hierarchical diffusion prevails initially across large towns only but emerges in the full spectrum of settlements in the middle of the life cycle. Interestingly, we discover a characteristic size difference as a sign of hierarchical diffusion at the end of the life cycle. Fixing the urban hierarchy variables at given levels, we find that the role of distance is minor in the beginning, intensifies in the middle, and shades away towards the end of the life cycle. This finding contradicts previous gravity estimations that have suggested monotonous intensification of distance effect.

Our results confirm that spatial diffusion of innovations occurs along the hierarchical order of cities. We provide new insights into the dynamics of the exact mechanism over the innovation life cycle. This new evidence is important for local adoption predictions that aim to overcome the systemic biases of settlement size and geographical distance in network diffusion models.

## 2. Results

### 2.1. Data

Our analysis is based on a dataset collected from iWiW, a Hungarian online social networking site launched in 2002. The service was a remarkable innovation in the Hungarian market of online services of its time and became highly popular in 2005. Almost 3 million profiles were registered until 2008 (30% of total population), when iWiW was the most visited Hungarian webpage. The life cycle of iWiW is depicted by monthly registrations in electronic supplementary material, S1, in which registrations are broken down to age groups as well. The website lost its primacy from 2010, when Facebook overtook the market, and the iWiW owner stopped the service in 2014. We have access to the anonymized version of all public profile data on iWiW, including friendship ties, demographics, location, registration date and date of last login. Data were collected and delivered by the data owner in January 2013. Previous research on iWiW has looked into the spatial pattern of the iWiW social network and its relation with socio-economic phenomena [22–24], churn from the website [25,26] and diffusion [20,27].

In this paper, we analyse invitation patterns that capture directed and geolocated spreading events at unprecedented scale and time horizon. Until 2012, users could only register on the website if they had been invited by another iWiW user. The individual-level data used in this paper contains the ID of iWiW users, the ID of those users who sent the invitation, self-reported location information of users at the settlement level and day of registration. Thus, we are able to construct a network of invitations from our dataset, where nodes are the iWiW users, and directed edges between them represent the inviter–invited relationships at the individual level. This way, we create a network with 3 059 363 users and 2 636 779 invitations between them. We then aggregate our individual-level network into the 2555 Hungarian settlements given in the user's town of residence field in each year of the life cycle. In this dynamic settlement-level spatial network, we have directed edges with an edge weight corresponding to the number of invitations going from the source settlement to the target settlement in the given year. We omit self-loops from the analysis, where the settlement of the source node and the target node are identical.

### 2.2. Overlap of hierarchical and neighbourhood diffusion

**Figure 1.**
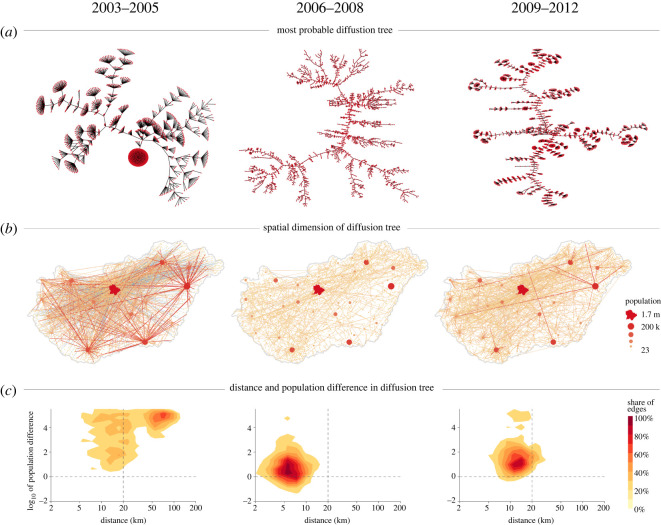
Hierarchical and neighbourhood diffusion over the innovation life cycle. (*a*) Most probable invitation paths given by the solution of the minimum weight branching problem on the transformed network with ${\tilde{\;p}}_{ST}$ weights. Nodes correspond to settlements. (*b*) Minimum weight invitation trees of the first row with settlements positioned on the map of Hungary. Edges are coloured according to the size of the source settlement (edges with Budapest as a source are blue), that is also indicated by the size of the nodes. (*c*) Distribution of tree edges with respect to two measures: distance between source and target settlements (horizontal axis), and log_10_ of source and target population size fraction (vertical axis). The vertical black line separates tree edges with less than 20 km distance between source and target, the horizontal black line separates tree edges that go down the settlement hierarchy (source size is larger than the target size), and that go up the settlement hierarchy (target size is larger than the source size). The top left quadrant corresponds to edges that are both sent to a very close distance and in a downwards hierarchical pattern.

To demonstrate the problem that hierarchical and neighbourhood diffusion often overlap, figure 1 illustrates the most probable invitation tree in the spatial diffusion measured from the aggregated settlement-level network across the iWiW life cycle. These settlement-level invitation networks consist of weighted directed edges of total invitation counts between settlements over three time periods: 2003–2005, 2006–2008 and 2009–2012. We denote the number of invitations in a given time period going from settlement *S* (source) to settlement *T* (target) by *w*_*ST*_. To identify most probable diffusion paths, we normalize *w*_*ST*_ by the number of total outgoing invitations such that edges represent outgoing invitation probabilities *p*_*ST*_, where $p_{ST}=w_{ST}/\sum_{T}w_{ST}$. The probability of a cross-settlement diffusion path is the product of these *p*_*ST*_ values along the path edges. We normalize weights by taking the logarithm and multiply by −1 so that the new weights are ${\tilde{\;p}}_{ST}=-\log(w_{ST}/\sum_{T}w_{ST})$. This transformation makes the maximum probability tree of the graph the one for which the sum of the weights is minimal. Using the modified weights enables us to search for the most probable invitation tree [28,29] by detecting the minimum weight branching in the invitation network [30]. We use the efficient implementation of the Chu–Liu–Edmonds algorithm for obtaining the minimum weight spanning tree by Gabow *et al.* [31].

Figure 1*a* reports a characteristic change in the pattern of major flows of diffusion across settlements. In 2003–2005, the paths in the tree are short such that leaves can be reached through only a low number of internal vertices. The tree even includes a node that has an especially large degree corresponding to Budapest that acts as a major source of invitations during this period. These observations suggest fast diffusion cascades, in which few settlements stand out in distributing the innovation. However, the pattern changes in 2006–2008 when paths in the diffusion tree form long chains, and large-degree nodes disappear, signalling a smoother and more equal diffusion through the whole size-spectrum of settlements. This tree with long paths remains in years 2009–2012 as well, but smaller cascades emerge again at the tip of the branches. Other measures derived from the full weighted settlement-level network also support these observations; table 1 shows the hierarchy measure, or global reaching centrality of [32], and average modularity scores of 10 partitionings obtained by the Louvain algorithm [33] for the three time periods. According to the global reaching centrality, the invitation network is least hierarchical and least modular in 2006–2008, and most hierarchical in 2003–2005.

**Table 1.** Global reaching centrality [32] and modularity [33] scores for the three aggregated settlement-level weighted networks.

network	2003–2005	2006–2008	2009–2012
global reaching centrality	1.264	0.501	0.665
modularity	0.271 ± 0.004	0.232 ± 0.005	0.318 ± 0.005

The spatial pattern of these diffusion trees, illustrated in figure 1*b*, provides support to the hierarchical diffusion of Hägerstrand [6]. Hierarchical diffusion of iWiW initially happened only from the largest cities that have mediated the innovation to all other settlements even to large distances. Later, from the middle of the life cycle, the long diffusion paths have emerged along the urban hierarchy such that flows of spreading went from slightly larger to slightly smaller settlements to a smaller geographical distance. Finally, at the end of the life cycle, both mechanisms contribute to the diffusion.

Next, we further demonstrate the co-evolution of the hierarchical and neighbourhood diffusion over the life cycle in figure 1*c*. This is done by plotting the geographical distance between settlements connected by the diffusion tree in figure 1*a* against the population difference (measured as the log_10_ of the fraction of the source and the target population sizes). The first period is characterized by long-distance cascades of hierarchical diffusion. This changes to local diffusion across similar settlements in the second period. Finally, the emerging small cascades remain local.

Taken together, in the above descriptive analysis we find by looking at the most probable diffusion tree across settlements that hierarchical diffusion and neighbourhood diffusion are closely related and co-evolve over the life cycle. Thus, their joint systematic analysis is necessary. electronic supplementary material, S2 further describes the dynamics of hierarchical and neighbourhood invitations.

### 2.3. Disentangling hierarchical and neighbourhood diffusion

According the observations presented above, the effect of urban hierarchy in spatial diffusion must be disentangled from other major geographical factors. The first of such geographical factors is the effect of the urban concentration captured by urban scaling law, and the second is the effect of distance captured by the gravity law, both of which have been previously found to characterize spatial diffusion of iWiW [20]. The scaling effect [34] manifests in disproportionally more invitations sent from large settlements early on in the life cycle compared with what their population size would linearly suggest, but this superlinear scaling disappears throughout the majority and the laggard phases [20]. The gravity effect captures the intensity of diffusion between settlements *S* and *T* as a function of populations *P*_*S*_ and *P*_*T*_ and the term *d*^−*χ*^, where *d* is the Euclidean distance between the two settlements, and *χ* is the gravity coefficient, which is found to have an increasing power on diffusion over the life cycle [20].

We apply a regression framework to capture the role of hierarchical diffusion, the effect that an innovation is more likely to go from larger settlements towards smaller settlements, assuming the co-presence of urban scaling of adoption and the gravity law in sending invitations. In every year, we observe the number of invitations going between settlement pairs *w*_*ST*_, but there are even more settlement pairs between which there is no invitation process at all. To account for these excess zero observations, we use a zero-inflated negative binomial regression model [35]. In this regression technique, we assume that the excess zero counts come from a logit model and the remaining counts come from a negative binomial model specified in Material and methods. We can predict invitation counts by including all possible settlement pairs into the data.

We estimate the following equation for the invitation counts *w*_*ST*_:

2.1 $$\begin{array}{rl} \log w_{ST} & \approx \alpha {\left(\log P_{S}\right)}^{2}+\beta \cdot (\log P_{S}\cdot \log P_{T})+\gamma {\left(\log P_{T}\right)}^{2}+\delta \log P_{S}+\varepsilon \log P_{T}\\ & \;+\chi \log d_{ST}+C, \end{array}$$

where the coefficients *α*, *β*, *γ*, *δ*, ɛ and *χ*, and the constant *C* are given by the maximum-likelihood estimator of the ZINB model in Stata [36]. To account for the gravity effect, we include the distance term log *d* and the population terms corresponding to the source and target populations log *P*_*S*_ and log *P*_*T*_. However, figure 1 suggests that the role of hierarchy changes in the life cycle, because the most probable tree of invitations shifts from larger towns to long hierarchy chains, then to smaller towns dominating the invitations. Therefore, we add second-order terms (log *P*_*S*_)^2^, (log *P*_*T*_)^2^ and log *P*_*S*_ · log *P*_*T*_ to the estimation, which can capture and test multiple hierarchical invitation scenarios by using terms that are able to describe a general second-order surface in the variables log *P*_*S*_ and log *P*_*T*_. For the full regression tables and the logit part of the model, see regression coefficients and parameters in the tables of electronic supplementary material, S3. For assessment of the goodness of fit of the models for each year, see electronic supplementary material, S4. Even though the ZINB model is more effective at predicting zero invitation counts, it performs reasonably well in reproducing invitation counts and their distribution.

We start with the effect of settlement size. We would like to understand how the source and target settlement populations influence invitation counts if we control for distance. In other words, given two settlement pairs for which the distance is equal, how does the invitation count differ for different *P*_*S*_ and *P*_*T*_ pairs. Because of the second-order terms in the regression, the surface defined by the population terms

$$\alpha {\left(\log P_{S}\right)}^{2}+\beta \cdot (\log P_{S}\cdot \log P_{T})+\gamma {\left(\log P_{T}\right)}^{2}+\delta \log P_{S}+\varepsilon \log P_{T}+C$$

is a general second-order surface. We are going to refer to these above terms as the contribution to log *w*_*ST*_, the logarithmic number of invitation between the source and target settlements coming from the terms containing the population values. For us, the important range from this surface that is defined by the regression coefficients is where both *P*_*S*_ and *P*_*T*_ are within realistic population sizes, that is, if they are larger than a small village (≈200 people), but smaller than the capital city Budapest (≈2 · 10^6^ people).

**Figure 2.**
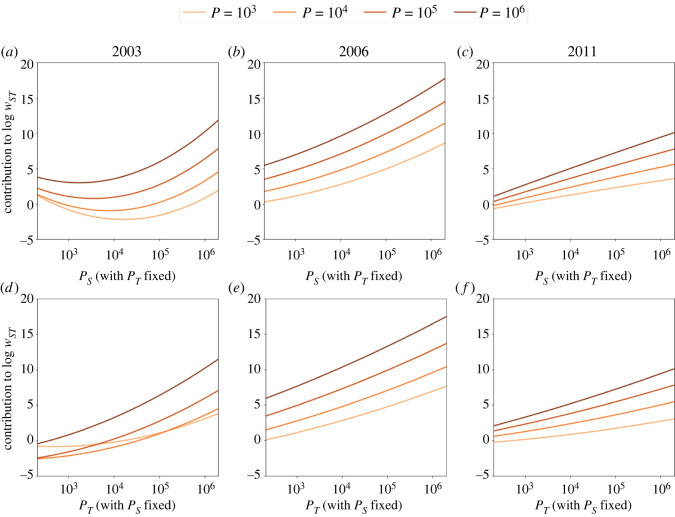
Settlement size in spatial diffusion. The effect of source (*a*–*c*) and target (*d*–*f*) settlement size controlling for all other variables in the three selected years 2003, 2006 and 2011.

First, we show how log *w*_*ST*_ changes with *P*_*S*_ for four different target population sizes *P*_*T*_ (figure 2*a*–*c*) in three selected years (2003, 2006 and 2011) that are in three different phases of the life cycle. These four different target populations roughly represent villages (10^3^), small towns (10^4^), larger towns (10^5^) and the magnitude of the capital city, Budapest (10^6^). In 2003, there is a minimum in the curves of all size regimes, meaning that mid-sized source settlements act less as inviters in the network than small villages and large towns. The contribution to the log invitation count is highest for large towns and the capital, and the relationship is significantly nonlinear between log *w*_*ST*_ and log *P*_*s*_ (as confirmed by the coefficients of second-order terms in electronic supplementary material, S3). This suggests that in the beginning of the life cycle of a product, the effect of source settlement population size is even larger than what an urban scaling law would predict. In 2006, log *w*_*ST*_ increases with log *P*_*S*_ in all four selected *P*_*T*_ regimes, therefore, the larger the source settlement, the more the invitation count for a given target size. In 2011, this monotonicity still holds, although the relationship becomes concave suggesting a faster-than-power-law decrease in the role of large settlements in the diffusion of invitations. For given source settlement sizes (again 10^3^, 10^4^, 10^5^ and 10^6^), panels *d*–*f* show that an increasing target settlement size increases invitation counts. The curves from *a*–*f* (as well as coefficients in electronic supplementary material, S4) show that second-order terms are indeed important in estimating the number of invitations; therefore, the observed relationships are rarely linear between log *w*_*ST*_ and log *P*_*S*_ or log *P*_*T*_. This means that power-laws between log *w*_*ST*_ and any combination of *P*_*S*_ and *P*_*T*_ are not feasible models for this system.

**Figure 3.**
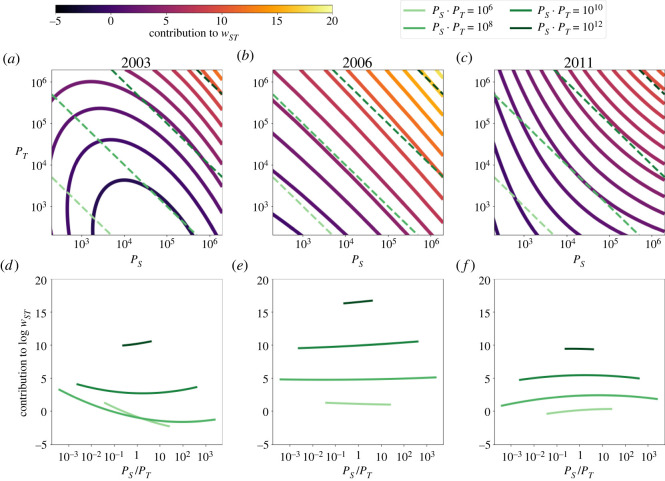
Urban hierarchy in spatial diffusion. Contour lines in panels (*a*–*c*) show level curves of the contribution of the source (*P*_*S*_) and target (*P*_*T*_) settlement sizes to the negative binomial term of the regression with controlling for distance *d*. Lighter colour indicates more invitations, see the colour bar at the top left corner. Green dashed lines correspond to (*P*_*S*_, *P*_*T*_) pairs where the number of potential total connections between two settlements is constant, that is, where *P*_*S*_ · *P*_*T*_ is constant, with darker colour referring to more potential total connections (10^4^, 10^6^, 10^8^ or 10^10^). Panels (*d*–*f*) show the surface height corresponding to the position of the green dashed lines from panels (*a*–*c*). These are the estimated invitation count contributions controlling for the settlement pair’s potential total contacts and distance.

But figure 2 does not tell us about the effect of hierarchy, since we cannot compare the combined effect and relative position of *P*_*S*_ and *P*_*T*_ on log *w*_*ST*_. Figure 3*a–c* presents the level curves of the contribution to log *w*_*ST*_ from the population terms and the constant *C* from equation (2.1) in the variable space of *P*_*S*_ and *P*_*T*_. Colouring of the level curves goes from the darkest minimum to the lightest maximum. In the bottom left corner of panels *a*–*c*, *P*_*S*_ and *P*_*T*_ both correspond to small village population sizes, whereas the top right corner corresponds to the case where both *P*_*S*_ and *P*_*T*_ are as large as Budapest, the capital city. In between, this area represents every possible (even hypothetical) settlement pair in Hungary in terms of *P*_*S*_ and *P*_*T*_. As expected, the number of invitations is highest in all three selected years in the top right corner: if both the source and the target settlements are very big. But the finer structure of this estimation is remarkable, and is able to tell us more about the hierarchy effects in detail.

As a most important marker of hierarchy effects, we investigate four different cross-sections of this surface estimated by ZINB that are marked by the green dashed lines of tangent −1 in the upper panels. These green dashed lines correspond to *P*_*S*_ · *P*_*T*_ = constant pairs, in other words, these lines mark all settlement pairs for which the potential number of all connections is the same. Along this line in panels *a–c*, in the very middle, there is the case when *P*_*S*_ = *P*_*T*_, but towards the right bottom corner, *P*_*S*_ > *P*_*T*_, and the population share of the source settlement grows. Towards the top left corner, *P*_*T*_ > *P*_*S*_, and the population share of the target settlement grows for the fixed number of total connections. The bottom panels *d–f* show the surface height along these selected lines, whose shape can be classified into four different categories.

1. If it is increasing towards large *P*_*S*_/*P*_*T*_ values, it means that the invitation contribution is larger for those pairs, in which the source settlement is larger than the target, even if there are the same number of total possible connections for other pairs. This corresponds to the hierarchical invitation pattern in which larger settlements tend to invite smaller settlements.
2. On the other hand, if this curve is monotonically decreasing, then it is exactly the other way round: relatively larger targets and smaller sources lead to more invitations. This corresponds to a reverse effect in which invitations flow back from smaller settlements to larger ones.
3. If there is a minimum in this curve within the given realistic size regimes, it means that larger size differences between *P*_*S*_/*P*_*T*_ are favoured in both directions compared with settlements of roughly the same sizes, or that large size differences in either direction lead to more invitations.
4. A maximum would select one ‘favoured’ size difference for which invitation count contribution is maximal.

We can see that the largest size regime always falls into the first category of the above four, here, a relatively larger source settlement in the pair yields more invitations (case 1). However, in 2003, the two smallest categories, *P*_*S*_ · *P*_*T*_ = 10^6^, and *P*_*S*_ · *P*_*T*_ = 10^8^ both show a strong inclination for reverse hierarchical invitations (case 2), where the smaller *P*_*S*_/*P*_*T*_ is, the larger the invitation count. And the regime of cities with roughly 100 000 population disfavours similar sizes: contribution is largest at both ends of the possible *P*_*S*_/*P*_*T*_ regime (case 4). In 2006, these remarkably diverging behaviours come close to each other, and in the two largest size regimes, there is a small hierarchical effect (case 1), the third line has a very shallow minimum, meaning that hierarchy plays almost no role here, and there is a very slight decreasing effect for the smallest settlement pairs (case 2). In 2011, the three largest curves show a favour for similar settlement sizes (case 3), and a slight hierarchical effect for the smaller settlement pairs (case 1).

**Figure 4.**
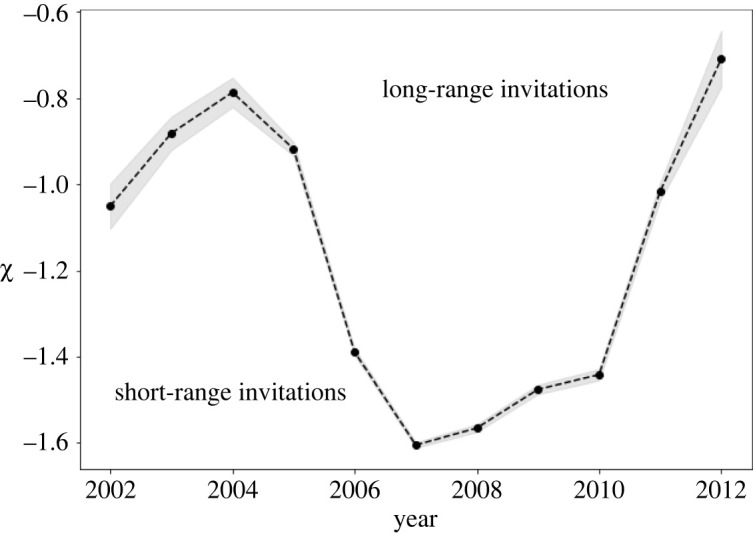
Distance effect in spatial diffusion. Value of the coefficient *χ* estimated from the negative binomial part of the ZINB model characterizing the dependence of invitations on spatial distance between two settlements.

Finally, figure 4 shows the coefficient *χ* of the distance term log *d*_*ST*_ from equation (2.1) with its estimated error. There is a clear distinction between years with more long-range invitations (2002–2005, 2011–2012), and the short-range invitation years (2006–2010). This result from the regression is thus in line with the intuitive picture from figure 1, in which the share of invitations sent to less than 20 km was very high in figure 1*c* for the most probable invitation tree edges in years 2006–2009. Here we can see that this effect remains if we control for excessive zero counts and population sizes.

## 3. Discussion

This paper contributes to the traditional discussion on spatial diffusion of innovations by disentangling the role of urban hierarchy from geographical distance using data from unprecedented detail and timescale. Looking at the most probable diffusion paths using direction and timestamp of nearly 3 million spreading events, we discover that diffusion initially cascades from large cities to all other settlements. Later, in the middle of the life cycle when adoption accelerates, hierarchical diffusion manifests in long chains and emerges along the entire spectrum of urban hierarchy. Unlike in previous research that investigated distance effect separately, our multivariate approach unfolds the real effect of geographical distance that intensifies stronger around the adoption peak but becomes weak again in the late phases of the life cycle.

Our results shed new light on the main puzzles of spatial diffusion research: how is the spatial distribution of individuals open to novelty and how does the structure of social interaction networks influence diffusion prediction at local scales? Previous models have found that settlement size and distance are remaining sources of error in complex contagion models. The finding that the role of urban hierarchy is dynamically changing over the life cycle and has a consequence on the role of distance as well might be important for future models. Simulated complex contagion models on social networks should incorporate the hierarchical diffusion processes.

The consequences of these findings might reach far concerning the potentials of policy interventions in supporting or blocking diffusion. Adoption of new technologies or scientific knowledge is the engine of progress; thus, a policy targeting adoption according to the place of the settlement in the urban hierarchy can benefit the society. However, the costs and motivations of adoption vary greatly across products and technologies; hence, the generality of spatial patterns reported here must be looked at across various innovation flows. The diffusion of social media is a specific example in which adoption is greatly motivated by social interactions on the website. To prove the generality of the illustrated spatial diffusion patterns, one needs to investigate products that are not strongly associated with the social dimension. Policy might want to block the diffusion of harmful novelty, such as fake news. However, these typically spread very quickly; thus, future research shall compare spatial diffusion of innovations of long and short life cycles. Finally, other socio-economic factors such as development, instrastructure, education and institutions, among others, might have a role in diffusion that we did not consider in this paper.

## 4. Material and methods

### 4.1. Data

Although free registration became possible in 2012, this previously singular option remained the major means of joining the website, with more than 60% of iWiW users registering through an inviter even after this date. Thus, for the majority of the users, we have the anonymized identifier of the inviting user as well. Since our data was collected in 2013, the ID of the inviter was missing in those cases when the profile of invitation sender was already deleted or the profile was registered after June 2012 via the free registration option.

Users had to choose a town of residence to be displayed along with their profiles. Users could change this location field easily, moreover, the reliability of this piece of information cannot be checked, although the menu offered a limited number of valid location strings for users. Nevertheless, we use this town of residence as geolocation information about both the users and their inviters.

In our original network, individual users are geolocated at the settlement level using the self-reported location field, we leave out users having more than one, but less than 5000 connections, and we omit users for whom the first and last login happened on the very same day. Moreover, we only include users with a Hungarian location, since a certain number of the registrations came from neighbouring or other European countries.

Individual data access is restricted by a non-disclosure agreement between the research group and the data owner. Aggregated data and code can be downloaded from the GitHub repository https://github.com/bokae/iwiw_invitations/.

We create aggregated invitation networks by grouping individual nodes into settlements, leading to directed edges between Hungarian settlements with an edge weight corresponding to the number of invitations going from the source settlement to the target settlement in a given year. We omit self-loops from the analysis, where the settlement of the source node and the target node are identical.

### 4.2. Most probable invitation paths

We construct the most probable spreading of the invitations on the settlement networks by looking for the most probable invitation tree using an efficient implementation of the Chu–Liu–Edmonds algorithm [31]. We suppose that the probability of an invitation path is the product of the probabilities along the constituting directed edges, where we normalize invitation counts by the number of total invitations going out of the source settlement in three time periods (2003–2005, 2006–2009, 2010–2012). By taking the logarithm and multiplying edge weights by −1, this problem is equivalent to finding the minimum weight branching in the directed weighted invitation network. Because the network is not always strongly connected, we add an artificial source node that we connect to every other node with a sufficiently large edge weight such that the algorithm avoids these edges if possible, but we remove the artificial root node at the end of the process.

### 4.3. Regression framework

The zero-inflated negative binomial regression model is formalized by:

4.1 $$w_{ST}=e^{k}\cdot \underset{\mathrm{non-zero inv. probability}}{\underbrace{\left(1-\frac{e^{l}}{1+e^{l}}\right)}},$$

4.2 $$\log w_{ST}=k+\underset{\mathrm{small}}{\underbrace{\log\left(1-\frac{e^{l}}{1+e^{l}}\right)}}.$$

In the first of the above equations (4.1), *l* is the log-odds term from the logit model that is used to estimate the probability that there is a non-zero number of invitations between settlement pairs. The parameter *k* characterizes the actual invitation counts between settlement pairs given that the count is non-zero. The second equation (4.2) shows that the contribution to log *w*_*ST*_ is mostly contained in the term *k*, since the probability of the invitation between the two settlements being non-zero is close to one, for which the logarithm is close to 0. Both *k* and *l* are governed by the processes mentioned before, therefore, we choose the terms that predict them in the ZINB model to reflect these.

We estimate the terms *k* and *l* from equation (4.1) for each year between 2002–2012 using Stata [36].

## Data Availability

Because individuals might be identified in the social media data owned by the telecommunication company, data tenure was controlled by a non-disclosure agreement between the owner of iWiW data, and the research group. The recent owner of the data is New Wave Media Ltd (contact: https://nwmgroup.hu). Raw data are not publicly available due to privacy considerations and are only used in an aggregated form in the analysis presented in this paper. Raw data are available to researchers who meet the criteria for access to confidential data, sign a confidentiality agreement and agree to work under supervision at the Centre for Economic and Regional Studies. Data access can be requested by email to Balázs Lengyel: lengyel.balazs@krtk.hu. Settlement-level aggregated data and all the code needed to reproduce the analysis and figures of the paper can be downloaded from the GitHub repository https://github.com/bokae/iwiw_invitations.
